# Sexual life and intimate relationship experience among bladder cancer patients: a systematic review and qualitative meta-synthesis

**DOI:** 10.3389/fpubh.2026.1887122

**Published:** 2026-08-19

**Authors:** Shuting Tan, Chunhua Nie, Suo Yang, Cong Dai, Yongheng Li

**Affiliations:** The Fourth Hospital of Changsha (Integrated Traditional Chinese and Western Medicine Hospital of Changsha), Changsha, China

**Keywords:** bladder cancer, experience, intimate relationship, meta-synthesis, sexual life

## Abstract

**Objectives:**

The global incidence of bladder cancer continues to rise. Treatments for this disease, particularly radical cystectomy and urinary diversion, can profoundly disrupt sexual function and intimate relationships, leading to physiological, psychological, relational, and existential changes. However, qualitative evidence syntheses that integrate these experiences remain limited. This review aimed to synthesize qualitative research on bladder cancer patients’ sexual life and intimate relationship experiences in order to inform the development of couple-centered interventions and continuous sexual rehabilitation services.

**Methods:**

This study was conducted as a systematic review and qualitative meta-synthesis and was reported in accordance with ENTREQ. PubMed, Web of Science, Embase, Cochrane Library, CINAHL, CNKI, WanFang, and VIP were searched from database inception to January 2026 for qualitative studies on the sexual life and intimate relationship experiences of bladder cancer patients. Studies were selected according to predefined PICoS-based eligibility criteria. Methodological quality was independently appraised by two reviewers using the Joanna Briggs Institute Critical Appraisal Checklist for Qualitative Research, and the findings were synthesized using Thomas and Harden’s three-stage thematic synthesis approach.

**Results:**

A total of 10 studies involving 183 participants were included in the synthesis. Fifty-four qualitative findings were aggregated into nine categories and then synthesized into three overarching findings: (1) multiple changes and challenges in sexual life after treatment; (2) changes and adjustments in intimate relationships; and (3) needs and expectations for sexual rehabilitation.

**Conclusion:**

Bladder cancer patients face complex physiological, psychological, relational, and existential challenges in their sexual lives and intimate relationships. Healthcare professionals should address these issues through holistic, couple-centered, and culturally sensitive care that supports both sexual recovery and long-term quality of life.

**Systematic review registration:**

https://www.crd.york.ac.uk/PROSPERO/view, identifier CRD420251027779.

## 1 Introduction

Bladder cancer ranks as the second most prevalent urological malignancy and the tenth most common cancer globally, with an estimated 549,000 new cases and over 200,000 fatalities annually (1). Bladder cancer is classified into two primary types: muscle-invasive bladder cancer (MIBC) and non-muscle-invasive bladder cancer (NMIBC). Approximately 25–30% of patients exhibit MIBC, whereas 70–75% present with NMIBC (2). In terms of geographical distribution, the incidence of bladder cancer is the highest in Africa, Southern and Eastern Europe, the Middle East, and North America (3). Moreover, The incidence of bladder cancer in males is approximately 3–4 times greater than in females and escalates with age, particularly among men aged 50 to 70 (4). By 2023, China is anticipated to report over 92,000 new bladder cancer cases, resulting in around 43,000 fatalities and a male-to-female ratio of 2.7: 1, imposing a substantial strain on national healthcare expenditures due to the necessity for extensive treatment and follow-up measures (5). Overall, the high incidence and mortality of bladder cancer underscore its major clinical burden and set the stage for considering the consequences of its standard surgical treatment.

Radical cystectomy accompanied by pelvic lymph node dissection and urine diversion constitutes the conventional therapy for surgically suitable individuals with non-metastatic muscle-invasive bladder carcinoma, and is furthermore advised for certain people with extremely high-risk non-muscle-invasive bladder cancer (6). This procedure, albeit offering considerable clinical benefits, is one of the most physically and mentally taxing interventions in urologic oncology (7). In males, standard radical cystectomy generally involves the extraction of the bladder, prostate, and seminal vesicles (8), whereas in females, it consists of bladder removal and may additionally encompass nearby reproductive organs and a portion of the anterior vaginal wall based on tumor positioning and oncological factors (9). Thus, bladder cancer patients must adapt to a novel urine diversion technique, often involving a urostomy, along with considerable anatomical, functional, and body-image changes following surgery. Previous studies demonstrate that radical cystectomy is associated with considerable postoperative morbidity and challenges in long-term survival, encompassing urinary and bowel dysfunction, stoma-related complications, concerns arising from urinary diversion, impaired body image, and psychological anguish (10). Sexual dysfunction is very common and clinically pertinent among these negative outcomes. Both male and female patients may experience considerable sexual dysfunction after radical cystectomy due to neurovascular injury, structural alterations, hormonal variations, and psychological stress, which can negatively affect intimate relationships, self-esteem, and overall quality of life (11). The data collectively suggest that, alongside achieving cancer control, greater emphasis should be placed on the sexual and psychological consequences of radical cystectomy, thereby providing a strong rationale for further exploration of patients’ experiences of sexual distress following surgery.

With increasing attention being paid to sexual distress among bladder cancer patients, a growing body of research has emerged in recent years focusing on the incidence, risk factors, and lived experiences of sexual distress in this population. Currently, research on the sexual discomfort of these patients predominantly relies on cross-sectional surveys and studies of contributing factors, which depict the actual circumstances of patients (12). Data indicates that between 20 and 90% of patients encounter varying levels of sexual distress following radical cystectomy, with discrepancies among research attributed to changes in assessment instruments, follow-up durations, and patient demographics (13). This discomfort adversely affects marital relationships, intimacy, and psychological wellbeing, perhaps enduring well into survivorship (14). The results of these quantitative research have identified specific elements affecting sexual experiences in bladder cancer patients; nevertheless, they are primarily restricted to predetermined items and provide a limited comprehension of how individuals individually perceive and manage these changes. Given that sexual distress is profoundly subjective and intertwined with patients’ emotional, relational, and cultural contexts, qualitative research may yield more profound insights into patients’ experiences, emotions, and needs concerning sexual distress, thereby serving as a crucial complement to current quantitative evidence.

An increasing number of qualitative studies are presently being undertaken to concentrate on the sexual distress experiences among bladder cancer patients. For instance, Ceasar et al. conducted individual qualitative telephone interviews with 40 women diagnosed with bladder cancer who underwent radical cystectomy, revealing the necessity to expand the comprehension of sexual health beyond sexual intercourse to include sexuality and self-pleasure among participants (15). Zhang et al. determined that patients’ sexual lives following radical cystectomy face multiple problems, highlighting the necessity for healthcare professionals to attend to the sexual health needs of patients, including the intricacies of personal relationships throughout rehabilitation (16). Nevertheless, results from individual qualitative studies are frequently limited by small sample sizes, particular cultural contexts, and diverse research focuses, rendering it challenging for any single study to comprehensively encapsulate the intricate and multifaceted nature of sexual distress encountered by bladder cancer patients. Consequently, a meta-synthesis of qualitative research is essential to amalgamate, contrast, and reinterpret findings across studies. This study seeks to cultivate a more comprehensive and nuanced understanding of the sexual distress experiences of bladder cancer patients, thereby offering robust evidence for clinical practice and future interventions.

## 2 Methods

### 2.1 Study design

This study was designed as a systematic review and qualitative meta-synthesis of bladder cancer patients’ sexual life and intimate relationship experiences. Meta-synthesis is well suited to qualitative health research because it enables interpretive integration of meaning-based findings rather than statistical pooling of outcomes (17). The review followed the Joanna Briggs Institute Manual for systematic reviews of qualitative evidence (18) and was reported in accordance with ENTREQ guidance (19). To strengthen reviewer reflexivity, the review team discussed their disciplinary backgrounds and assumptions before data extraction and synthesis, kept analytic notes during coding, and resolved interpretive disagreements through team discussion with a third reviewer when needed. The study protocol was prospectively registered in PROSPERO (CRD420251027779).

### 2.2 Search strategy

The search strategy was prespecified by the research team and developed with input from a university medical librarian. PubMed, Web of Science, Embase, Cochrane Library, CINAHL, CNKI, WanFang, and VIP were searched from database inception to 31 January 2026. A combination of controlled vocabulary terms and free-text keywords related to bladder cancer, sexuality, intimate relationships, and qualitative research was used in each database, and the full search strings are reported in Supplementary File 1. In addition, the reference lists of all included studies were hand-searched to identify potentially relevant articles. Only studies published in English or Chinese were included.

### 2.3 Inclusion and exclusion criteria

Studies were eligible for inclusion if they met the PICoS-based criteria (20): (1) participants: adults aged 18 years or older with a confirmed diagnosis of bladder cancer; (2) phenomenon of interest: experiences, perceptions, feelings, or needs related to sexual life and intimate relationships; (3) context: any care setting, including hospital, community, home, or other healthcare settings; and (4) study design: qualitative studies or the qualitative component of mixed-methods studies published in English or Chinese. Studies were excluded if they were duplicate publications, conference abstracts, study protocols, posters, unavailable in full text, or contained incomplete data for which additional information could not be obtained from the authors.

### 2.4 Study selection

Following the searches, all retrieved records were imported into EndNote X9 and duplicate citations were removed. The remaining records were then exported to Excel, where two reviewers independently screened titles and abstracts and subsequently assessed full texts for eligibility. Regular meetings were held to resolve disagreements, and any unresolved discrepancy was adjudicated by a third reviewer. To minimize the risk of duplicate data inclusion, we additionally compared author lists, institutions, recruitment settings, study periods, and sample descriptions across potentially overlapping reports before final inclusion.

### 2.5 Quality appraisal

The methodological quality of each included study was independently appraised by two reviewers trained in evidence-based methodology using the Joanna Briggs Institute Critical Appraisal Checklist for Qualitative Research (21). The checklist contains 10 items rated as yes, no, unclear, or not applicable. Conflicts were settled via dialogue and, when required, by seeking the input of an external evaluator. No study was excluded solely on the basis of methodological quality, because our aim was to retain potentially valuable experiential data while accounting for reporting limitations in the confidence assessment.

### 2.6 Data extraction and synthesis

Two reviewers independently extracted key descriptive information from the included studies using a prespecified data-extraction form that captured author, publication year, country, participants, setting, aim, method, and findings. Extracted data were cross-checked for accuracy and completeness before synthesis.

Thomas and Harden’s three-stage approach was used for thematic synthesis by two reviewers working independently and then comparatively (22). Initially, pertinent excerpts from the findings sections of the incorporated research encompassing participant quotations and the authors’ analytical conclusions was imported into NVivo 11 and coded line by line. Second, codes with similar meaning were grouped inductively to construct descriptive categories. Third, these categories were compared across studies and progressively synthesized into higher-order analytical themes. Throughout this process, differences related to treatment modality, gender, relationship context, and survivorship stage were preserved during coding and then examined comparatively during theme development so that the synthesis remained interpretive rather than merely narrative. Any disagreement was resolved through consultation with a third reviewer.

### 2.7 Confidence in the findings

The ConQual approach was used to assess confidence in each synthesized finding (23). Following JBI guidance, dependability was evaluated primarily on the basis of methodological congruence items in the appraisal tool, and credibility was judged according to whether the supporting findings were unequivocal, equivocal, or unsupported. Each synthesized finding started at a high confidence level and was downgraded when methodological or reporting limitations warranted reduced confidence. The rationale for each downgrade is presented in Table 1 and further explained in the Results section.

**Table 1:** ConQual summary of findings for the qualitative meta-synthesis.

Synthesized finding	Dependability	Credibility	Initial rating	Reasons for downgrading	Final ConQual score
Synthesized Finding 1: Multiple changes and challenges in sexual life after treatment	Downgraded one level	Not downgraded	High	Several included studies did not adequately address the influence of the researcher on the research process, or the cultural/theoretical positioning of the researcher (JBI items Q1, Q6, Q7).	Moderate
Synthesized Finding 2: Changes and adjustments in intimate relationships	Downgraded one level	Not downgraded	High	Insufficient reporting of researcher positioning and reflexivity in some included studies (JBI items Q1, Q6, Q7).	Moderate
Synthesized Finding 3: Needs and expectations for sexual rehabilitation	Downgraded one level	Not downgraded	High	Insufficient reporting of researcher positioning and reflexivity in some included studies (JBI items Q1, Q6, Q7).	Moderate

## 3 Results

### 3.1 Search outcomes

**Figure 1:**
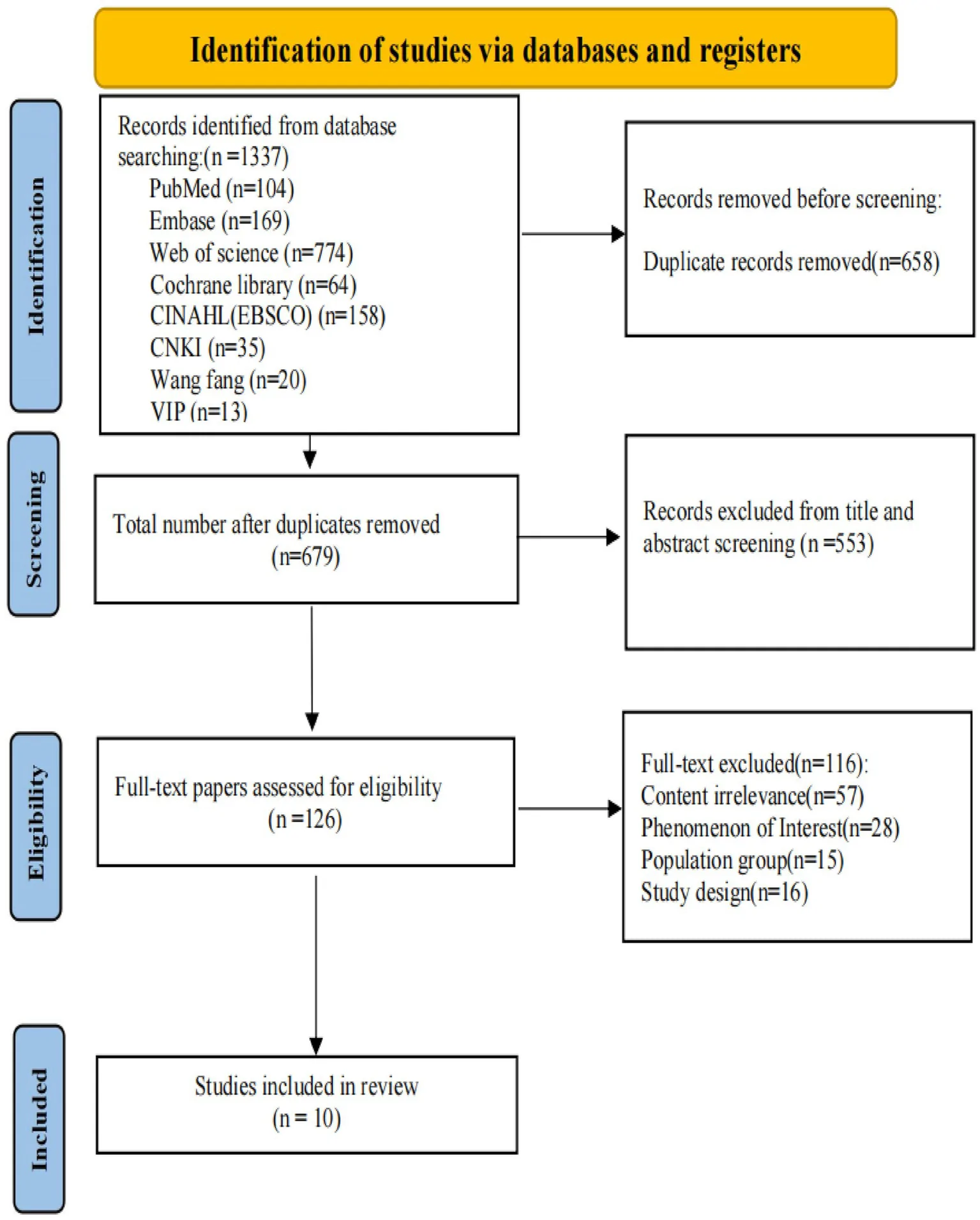
Flowchart of the identification and selection of studies.

As illustrated in Figure 1 1,337 records were identified through the database searches. After duplicate removal and title/abstract screening, 126 articles remained for full-text review. Figure 1 also details the reasons for exclusion at the full-text stage. Ultimately, 10 studies were included in this systematic review and qualitative meta-synthesis (15, 16, 24–31).

### 3.2 Characteristics of the included studies

Table 2 summarizes the key characteristics of the 10 studies included in this meta-synthesis. These studies were published between 2017 and 2026 and were conducted in the United States, China, Australia, Turkey, and Sweden. The included populations were intentionally heterogeneous and encompassed women and men, radical cystectomy recipients, TURBT survivors, patients living with urostomy or cutaneous ureterostomy, veterans, and patient-partner dyads across different survivorship time points. In a quantitative review, such heterogeneity would limit direct comparability of outcomes. In a qualitative meta-synthesis, however, it broadens the experiential range available for interpretation. Accordingly, our aim was not to assume identical outcomes across these groups, but to synthesize shared meanings and recurring patterns while preserving clinically relevant differences related to treatment modality, gender, and relational context.

**Table 2:** Characteristics of the included studies (*n* = 10).

Authors	Year	Country	Study design	Data collection	Participants	Phenomenon of interest	Main findings
Ceasar et al. (15)	2024	United States	Grounded theory approach	One-to-one telephone interviews	40 women who had undergone RC within the previous 6 months to 5 years	Women’s perceptions of sexual function and dysfunction after RC	Four themes: (1) marked informational deficits; (2) substantial physical and psychological barriers to sexual activity (3) disappointment when postoperative sexual experiences differed from preoperative expectations; (4) potential benefit from pelvic-floor or physical therapy.
Zhang et al. (16)	2026	China	Qualitative study integrating grounded theory and phenomenology	Semi-structured interviews	15 BC patients after RC	Sexual distress after RC for BC treatment	Four themes: (1) pronounced indicators of sexual discomfort; (2) adverse emotional conflicts; (3) various alterations in conjugal dynamics; (4) need for sexual rehabilitation
Zhang et al. (24)	2025	China	Phenomenological qualitative study	Semi-structured interviews	12 patients with BC undergoing cutaneous ureterostomy	Couples’ disease communication experiences after urinary diversion	Four themes: (1) avoidance of emotionally sensitive topics; (2) avoidant communication has adverse effects on patients; (3) experienced post-traumatic growth and gained positive communication experiences; (4) desire for professional communication support.
Heyes et al. (25)	2020	Australia	Qualitative descriptive study	Semi-structured interviews	10 patients with BC and their partners	Disruptions in quality of life for those diagnosed with breast cancer and their supporting partners.	Four themes: (1) bodily disruption; (2) cognitive reactions; (3) emotional responses; (4) everyday survival strategies
Gupta et al. (26)	2021	United States	Qualitative descriptive study	Semi-structured interviews and one focus group	25 participants (22 women and 3 partners) across pre- and post-RC cohorts	Psychosocial and sexual health concerns among women undergoing RC	Four themes: (1) altered body image; (2) psychological distress; (3) impaired sexual function; (4) inadequate clinician-led counselling regarding sexual health before and after surgery.
Kandemir et al. (27)	2017	Turkey	Phenomenological qualitative study	Semi-structured interviews	10 BC patients with urostomy and their partners	Sexual problems after urostomy for BC	Four themes: (1) body-image disruption; (2) practical difficulties during sexual activity; (3) insufficient access to professional support for couples; (4) partners reaction to problems
Caloudas et al. (28)	2026	United States	Qualitative interview study	Semi-structured interviews	14 male U. S. veteran BC survivors	Sexual problems and intimacy in long-term BC survivorship	Two themes: (1) sexual problems have adverse effects on intimate relationships; (2) unmet needs for better education, treatment support, and interdisciplinary survivorship care.
Löfgren et al. (29)	2023	Sweden	Qualitative interview study with content analysis	Individual interviews	10 women interviewed a median of 24 months after RC	Women’s experiences of sexuality after RC	Five themes: (1) feeling of intimacy; (2) the importance of the relationship; (3) hesitance to participate in sexual intercourse; (4) partner’s incapacity to partake in sexual activity; (5) acting for sexual recovery.
Yang et al. (30)	2025	China	Phenomenological qualitative study	Semi-structured in-depth interviews	15 BC patients after RC	Sexual distress, difficulties, and care needs after RC	Four themes: (1) prominent sexual dysfunction; (2) negative emotional consequences; (3) altered marital relationships; (4) a clear need for individualized sexual rehabilitation and psychological support.
Wang et al. (31)	2021	China	Qualitative study	Semi-structured in-depth interviews	32 men after TURBT for BC	Sexual experiences and rehabilitation needs after TURBT	Five themes: (1) intentional sexual restraint; (2) fear of harming their partners; (3) neglected sexual concerns; (4) negative emotional reactions; (5) expectations for proactive support from healthcare professionals.

BC = bladder cancer; RC = radical cystectomy; TURBT = transurethral resection of bladder tumor.

### 3.3 Quality appraisal of the included studies

The methodological quality of the 10 included studies was independently assessed by two reviewers using the JBI Critical Appraisal Checklist for Qualitative Research. As shown in Table 3, the appraisal scores ranged from 7 to 8 out of 10, indicating that all included studies were of moderate-to-high methodological quality. The most common limitations involved insufficient reporting of the researcher’s philosophical perspective, positioning, and reflexivity. No study was excluded based on quality appraisal, and all 10 studies were ultimately included in the meta-synthesis.

**Table 3:** Methodological quality appraisal of the included studies (*n* = 10).

Study	Q1	Q2	Q3	Q4	Q5	Q6	Q7	Q8	Q9	Q10	Score (yes/10)
Ceasar et al. (15)	Y	Y	Y	Y	Y	U	U	Y	Y	Y	8
Zhang et al. (16)	Y	Y	Y	Y	Y	U	U	Y	Y	Y	8
Zhang et al. (24)	Y	Y	Y	Y	Y	U	N	Y	Y	Y	8
Heyes et al. (25)	U	Y	Y	Y	Y	U	U	Y	Y	Y	7
Gupta et al. (26)	U	Y	Y	Y	Y	U	U	Y	Y	Y	7
Kandemir et al. (27)	Y	Y	Y	Y	Y	U	N	Y	Y	Y	8
Caloudas et al. (28)	U	Y	Y	Y	Y	U	U	Y	Y	Y	7
Löfgren et al. (29)	U	Y	Y	Y	Y	U	U	Y	Y	Y	7
Yang et al. (30)	Y	Y	Y	Y	Y	U	N	Y	Y	Y	8
Wang et al. (31)	Y	Y	Y	Y	Y	U	N	Y	Y	Y	8

C1: Is there alignment between the stated philosophical perspective and the research methodology? C2: Is there alignment between the research methodology and the research question or objectives? C3: Is there alignment between the methodology and the data collection methods? C4: Is there alignment between the methodology and the representation and analysis of data? C5: Is there alignment between the methodology and the interpretation of results? C6: Is there a statement locating the researcher culturally or theoretically? C7: Is the influence of the researcher on the research, and vice versa, addressed? C8: Are participants and their voices adequately represented? C9: Is the research ethical according to current criteria, or is there evidence of ethical approval? C10: Do the conclusions drawn in the research report flow from the analysis or interpretation of the data? Y, yes; N, no; U, unclear; NA, not applicable.

### 3.4 Qualitative meta-synthesis

**Figure 2:**
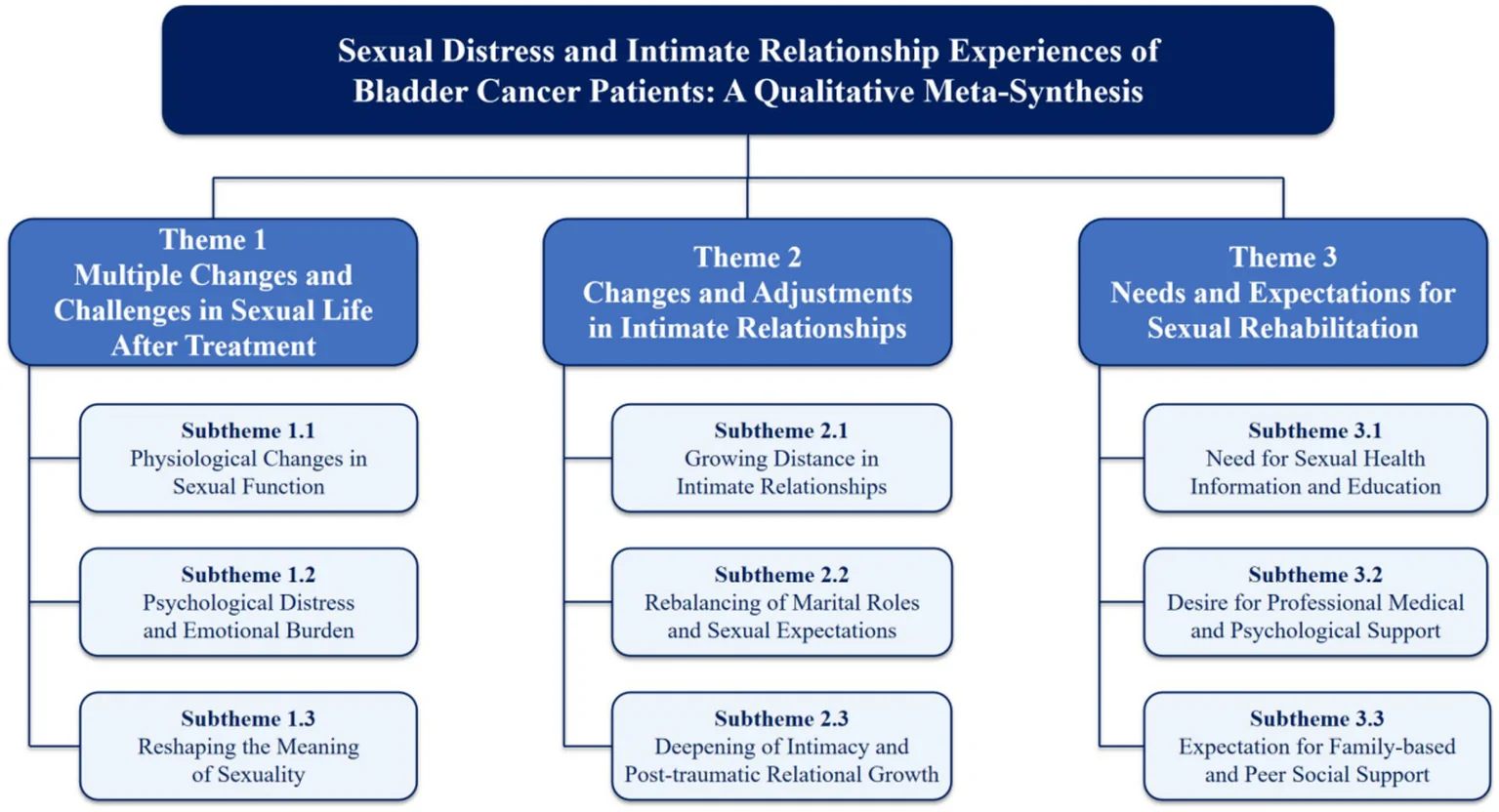
Theme and subtheme structure.

A total of 54 findings were extracted from the 10 included qualitative studies. Through iterative coding, comparison, and abstraction, these findings were grouped into nine categories and then synthesized into three higher-order findings, as illustrated in Figure 2: (1) multiple changes and challenges in sexual life after treatment; (2) changes and adjustments in intimate relationships; and (3) needs and expectations for sexual rehabilitation. The synthesis was interpretive rather than aggregative in a statistical sense, and it sought to identify cross-cutting experiential patterns while retaining important differences in treatment pathway, gender, and survivorship context.

#### 3.4.1 Theme 1: Multiple changes and challenges in sexual life after treatment

This synthesized finding was based on 21 findings grouped into three categories and describes the obstacles bladder cancer patients face in adjusting to altered sexuality after treatment. Across radical cystectomy, TURBT, urinary diversion, and survivorship settings, the findings consistently showed physiological disruption of sexual function, substantial psychological distress related to sexuality, and the need to reconstruct the meaning of sexuality in daily life. Rather than implying that all treatment groups experience identical outcomes, this theme captures shared experiential patterns that emerged across clinically different populations.

##### 3.4.1.1 Category 1: Physiological changes in sexual function

Following radical cystectomy or transurethral resection of bladder tumor, patients commonly reported physiological changes in sexual function, including erectile dysfunction, reduced desire, shorter or less frequent intercourse, and, in some cases, the cessation of sexual activity. Male participants frequently described erectile and ejaculatory difficulties [“After the surgery, I just could not get an erection anymore, no matter how hard I tried” (31)], whereas female participants reported vaginal shortening, dryness, narrowing, and dyspareunia following the removal of the uterus and anterior vaginal wall [“I think my vagina is still there? It just does not feel the same anymore” (15)]. Stoma-related discomfort, urinary leakage, and anxiety about the urostomy bag during intimacy further constrained sexual activity [“Every time we tried, I kept worrying about the bag—it just killed the moment” (27)]. Patients also noted that treatment-related comorbidities, postoperative discomfort, and fatigue reduced their physical capacity for sexual activity (16, 26).

##### 3.4.1.2 Category 2: Psychological distress and emotional burden

These physiological changes were closely intertwined with psychological distress. Many patients described anxiety, sadness, embarrassment, frustration, reduced self-esteem, and a pervasive sense of loss [“It’s killed my sex life—it’s killed who I used to be” (28)]. Some avoided sexual activity because of fear of cancer recurrence, concern about injuring their partner, or anxiety about sexual failure [“I’m scared that if we try and I cannot perform, it will only make things worse” (31)]. Body-image disturbance related to the stoma was particularly distressing for both men and women [“I do not even want to look at myself in the mirror, let alone let him see me like this” (26)]. Younger patients often mourned the loss of fertility and aspects of gender identity (16, 30), whereas some older patients described a more resigned form of acceptance, sometimes characterizing sexuality as something that had been “killed off” rather than openly grieved (25, 28).

##### 3.4.1.3 Category 3: Reshaping the meaning of sexuality

Across several studies, patients moved beyond a narrow, intercourse-centred understanding of sexuality and gradually redefined it to include intimacy, affection, touch, companionship, and emotional closeness [“Sex is not only about intercourse anymore—being held, being close, that means just as much now” (29)]. Women in particular described relying on non-penetrative forms of intimacy, such as cuddling, kissing, and sensual touch, to preserve closeness with their partners [“We’ve found new ways to be close—it’s different, but it’s still us” (15)]. Although this shift did not eliminate distress, it reflected an important adaptive process through which patients and couples maintained relational meaning despite physical limitations (16, 26, 28).

#### 3.4.2 Theme 2: Changes and adjustments in intimate relationships

This synthesized finding was derived from 16 findings grouped into three categories and describes how intimate relationships changed and were renegotiated after bladder cancer treatment. The findings showed that treatment did not simply affect sexual function in isolation; it also reshaped couple communication, partner roles, expectations of intimacy, and opportunities for relational growth. These relational changes appeared across heterogeneous samples, but their specific expression varied according to gender, treatment pathway, partner involvement, and sociocultural context.

##### 3.4.2.1 Category 4: Growing distance in intimate relationships

In some studies, sexual change was accompanied by communication breakdown and emotional distancing between patients and their partners. Patients often avoided discussing their feelings, sexual difficulties, or fears of death and recurrence [“We just did not talk about it—it was easier that way, but it also made me feel further away from him” (24)]. Such avoidant communication intensified alienation, undermined self-confidence, and weakened patients’ ability to cope with illness-related stress (16, 25, 30). In several accounts, partners responded with coldness, withdrawal, or rejection, which deepened patients’ shame and sense of being unwanted [“He did not touch me the same way anymore—sometimes I felt like he was already gone” (27)].

##### 3.4.2.2 Category 5: Rebalancing of marital roles and sexual expectations

Many couples actively renegotiated their sexual dynamics and marital roles after surgery. Partners often assumed caregiving responsibilities, while patients adjusted their expectations of intercourse and embraced alternative forms of closeness [“He helped me change the bag, he took care of me—in a way, we were closer than before, just in a different way” (27)]. Reciprocal compromise, role adjustment, and shared decision-making were described as essential for maintaining relational stability (16, 26, 29, 30). Patients also reported that open communication with their partners reduced psychological stress and restored a sense of balance in the relationship [“Once we started really talking about it, it felt like we were on the same team again” (31)].

##### 3.4.2.3 Category 6: Deepening of intimacy and post-traumatic relational growth

A substantial number of patients described post-traumatic growth within their relationships, suggesting that the shared experience of cancer sometimes strengthened the marital bond [“After all of this, I thought we actually love each other more—we have been through something most people never have to face” (25)]. Partners’ empathy, patience, and reassurance were central to emotional reconnection (16, 29). Shared coping also fostered camaraderie and mutual commitment [“Going through cancer together has changed us—we were more of a team now than we have ever been” (30)]. Taken together, these accounts suggest that, after an initial period of strain, adversity may in some cases deepen communication, affection, and resilience rather than diminish intimacy (24).

#### 3.4.3 Theme 3: needs and expectations for sexual rehabilitation

This synthesized finding was derived from 17 findings grouped into three categories and describes the needs and expectations of bladder cancer patients regarding sexual rehabilitation. Across the included studies, patients reported major information gaps and a strong need for professional medical and psychological support, as well as couple- and family-based social support. Together, these findings underscore the need for proactive, individualized, and multidisciplinary sexual rehabilitation pathways embedded within routine bladder cancer survivorship care.

##### 3.4.3.1 Category 7: Need for sexual health information and education

Across several studies, patients consistently reported inadequate knowledge about treatment-related sexual consequences and available rehabilitation strategies. Many stated that they had received little or no counselling before or after surgery [“Nobody ever told me sex would feel like this afterwards—I had to figure it all out myself” (15)]. They expressed a strong need for timely, accurate, and practical information about when and how sexual activity might be resumed, what bodily changes to expect, and what options might support recovery [“I wished someone had explained what to expect—even just a leaflet or a website, anything” (28)]. Patients also emphasized the value of receiving information through multiple channels, including written materials, digital resources, and direct discussion with clinicians or peers (16, 26).

##### 3.4.3.2 Category 8: Desire for professional medical and psychological support

Patients expressed a strong need for proactive, individualized, and interdisciplinary professional support for sexual rehabilitation. They expected healthcare professionals to initiate conversations about sexual health rather than waiting for patients to raise the issue [“If the doctor brought it up first, I think I could actually talk about it—but they never do” (26)]. The support they requested included input from urologists, oncology nurses, sex therapists, psychologists, and rehabilitation specialists, together with routine assessment of sexual concerns during follow-up (15, 16, 30, 31). Patients also highlighted the need for psychological counselling to address anxiety, sadness, body-image concerns, and fear of intimacy, particularly during the 6–12 months after surgery, when longer-term adjustment difficulties often became more apparent [“It was not right after the surgery—it was months later, when I realized this was my new normal, that I really needed someone to talk to” (28)].

##### 3.4.3.3 Category 9: Expectation for family-based and peer social support

Sexual rehabilitation was consistently framed as a relational rather than purely individual process. Patients valued partner-inclusive counselling and wanted their spouses to be involved in education, communication training, and decision-making [“I wished someone could talk to both of us together—he did not really know what I’m going through either” (24)]. Beyond partner involvement, patients also identified peer support from other bladder cancer survivors, family understanding, and broader social acceptance as important resources [“Talking to someone who had actually been through it made me feel less alone” (29)]. Online communities and survivor groups were viewed as safe spaces for sharing experiences and obtaining support, especially for issues that patients found difficult to discuss face to face (16, 25, 26, 30).

### 3.5 ConQual summary of the synthesized findings

Table 1 presents the ConQual summary of the synthesized findings. In this meta-synthesis, all 10 included studies clearly described their qualitative aims, data collection, and analytic procedures, but several studies provided limited reporting on researcher positioning, cultural/theoretical location, and reflexive influence (items C1, C6, and C7). For this reason, dependability was downgraded by one level for each synthesized finding. In contrast, the findings supporting the three synthesized conclusions were predominantly based on unequivocal participant quotations and clear interpretive links to the data; therefore, no downgrade in credibility was applied. The final ConQual rating for each synthesized finding was moderate, indicating that the evidence offers a meaningful basis for understanding sexual distress and intimate relationship experiences among bladder cancer patients, while also highlighting the need for stronger reflexive reporting in future qualitative studies.

## 4 Discussion

This meta-synthesis integrated qualitative evidence from 10 studies and generated three synthesized findings that illuminate the complex nature of sexual distress and intimate relationship experiences among bladder cancer patients. The findings indicate that postoperative sexual distress is not merely a biomedical consequence of treatment, but a broader survivorship challenge shaped by bodily change, psychological response, relational dynamics, and gaps in healthcare delivery. They also show that support from partners, clinicians, and peers is crucial in helping patients adapt, reconstruct meaning, and maintain quality of life. Taken together, the synthesis provides an evidence-informed foundation for improving sexual rehabilitation and survivorship care in bladder cancer.

### 4.1 Addressing multidimensional changes in sexual life after treatment

Synthesized Finding 1 indicates that bladder cancer patients experience substantial physiological, psychological, and existential changes in their sexual lives after treatment. Radical cystectomy frequently results in erectile dysfunction in men, with reported rates ranging from 80 to 90% in some studies (32), and may also cause vaginal shortening, dryness, narrowing, and dyspareunia in women because of the resection of gynecologic and vaginal structures (33). Even less invasive procedures such as TURBT can be associated with persistent sexual concerns and rehabilitation needs (34). Because this review is a qualitative meta-synthesis rather than a quantitative comparison of outcomes, these findings should not be interpreted as demonstrating equivalence between treatment groups. Instead, they show that different bladder cancer pathways can converge in producing sexual disruption, albeit through partly distinct mechanisms. Beyond physiological change, the present synthesis indicates that sexual distress is strongly mediated by psychological factors, including anxiety, depression, shame, fear of recurrence, fear of harming the partner, and body-image disruption related to urostomy or stoma (35). Maslow’s hierarchy of needs suggests that sexuality functions not only as a physiological need but also as a channel for intimacy, identity, and self-worth (36). The PLISSIT and EX-PLISSIT frameworks further imply that healthcare professionals need graded, permission-giving, and context-sensitive strategies for sexual communication rather than waiting for patients to raise concerns spontaneously (37). Taken together, these perspectives support a biopsychosocial interpretation of sexual recovery in which symptom burden, self-image, relational confidence, and meaning reconstruction must all be addressed in practice.

### 4.2 Recognizing the central role of intimate relationships in sexual recovery

Synthesized Finding 2 underscores that intimate relationships are central to patients’ adaptation to sexual change after treatment. Partners may become more distant, renegotiate roles, or develop deeper intimacy over time. This relational diversity is well explained by dyadic coping theory, which conceptualizes cancer as a “we-disease” in which both partners appraise, communicate about, and manage illness-related stress together (38). Dysfunctional dyadic coping, including avoidant communication, mutual withdrawal, or stigmatizing reactions, can intensify sexual difficulties and relational strain (39). In contrast, collaborative coping, open discussion, and joint problem-solving may strengthen emotional connection and improve sexual adjustment (40). Importantly, these couple processes are also shaped by cultural norms. In settings where sexuality is treated as private, shameful, or inappropriate for open discussion, couples may be especially likely to remain silent about distress, which can delay support-seeking and intensify misunderstanding. Thus, communication difficulties should be understood not only as interpersonal problems but also as culturally mediated patterns that influence how intimacy is negotiated after bladder cancer treatment.

Clinically, these findings suggest that sexual rehabilitation in bladder cancer should be reconceptualized as a couple-level rather than exclusively patient-level intervention. Intimate relationships are a core, and often overlooked, component of recovery after treatment; therefore, partners should be involved in sexual-health consultations from an early stage whenever this aligns with patient preferences, autonomy, and confidentiality. Couple-focused communication interventions may help dyads address sensitive issues such as altered body image, sexual anxiety, and the changing meaning of intimacy. Approaches such as therapist-facilitated dialogue, structured workshops, and expressive writing may reduce avoidance, improve mutual disclosure, and lessen the risk that partner withdrawal is misinterpreted as rejection. In addition, dyadic coping frameworks deserve greater integration into psycho-oncological care. Bodenmann’s Systemic-Transactional Model offers a useful basis for interventions that promote reciprocal stress communication, collaborative problem-solving, and mutual emotional regulation, including couple-based cognitive-behavioural therapy or sexuality-focused counselling (41). For dyads at heightened risk of relational decline, early screening with validated tools such as the Dyadic Adjustment Scale or the Dyadic Coping Inventory may facilitate timely referral to oncology-informed couples or sex therapy services (42). Overall, repositioning sexual rehabilitation as a dyadic component of routine bladder cancer survivorship care may strengthen sexual adjustment, relational resilience, and long-term quality of life.

### 4.3 Building an integrated, individualized sexual rehabilitation support system

Synthesized Finding 3 indicates a persistent mismatch between patients’ need for sexual rehabilitation and the support currently provided in routine care. Patients frequently reported inadequate knowledge about treatment-related sexual consequences, limited access to specialized support, and perceived hesitancy among clinicians to initiate sexual-health discussions. These findings can be meaningfully interpreted through person-centred survivorship care and the PLISSIT/EX-PLISSIT framework, both of which emphasize anticipatory communication, individualized education, and graded specialist referral (43, 44). They also align with broader international evidence showing that sexual counselling remains insufficient in bladder cancer care despite the high prevalence of sexual dysfunction in this population (45). Professional guidelines, including those from the European Association of Urology and the American Urological Association, support preoperative counselling and consideration of sexual-function-preserving strategies, but implementation remains uneven across clinical contexts (46).

Patients’ reported needs encompassed three interconnected dimensions of support: informational support (timely, clear, and personalized education regarding sexual recovery), medical and psychological support (proactive provider-initiated dialogue, multidisciplinary expertise, and focused psychosocial care), and social support (partner engagement, peer-survivor interaction, and family understanding). This structure is consistent with contemporary person-centred cancer survivorship guidance, which recommends that sexual health be addressed across the entire care continuum rather than only in response to overt complaint (47). However, sexual silence is reinforced by cultural taboo, embarrassment, limited privacy, time constraints, and insufficient professional training, particularly in contexts where open discussion of sexuality remains socially constrained (48, 49).

To address these gaps, several practical strategies are warranted. First, sexual-health counselling should be integrated into standard bladder cancer care from diagnosis through long-term follow-up rather than being reserved only for patients who explicitly request help (50). Second, multidisciplinary rehabilitation teams involving urologists, oncology and stoma-care nurses, psychologists, sex therapists, and pelvic-floor physiotherapists should be organized to provide individualized support tailored to age, gender, treatment modality, relationship status, and cultural background (51). Third, digital and peer-based resources may help reduce embarrassment and improve access to support for underserved groups (52). Overall, a structured and culturally sensitive rehabilitation pathway is needed to normalize sexual health as a legitimate and essential component of survivorship care.

### 4.4 Strengths and limitations

This meta-synthesis has several strengths. It is among the first qualitative evidence syntheses to focus specifically on sexual distress and intimate relationship experiences among bladder cancer patients while including women and men, Eastern and Western settings, and both radical and less invasive treatment trajectories. The use of the JBI Critical Appraisal Checklist and the ConQual approach further strengthens methodological transparency. Several limitations should also be acknowledged. First, only studies published in English and Chinese were included, which may have introduced language bias. Second, the included studies were heterogeneous with respect to sex, treatment modality, urinary diversion, and survivorship stage; although this heterogeneity enriched the interpretive range of the synthesis, it may limit the transferability of subgroup-specific insights. Third, cultural variation in sexual norms, marital expectations, and disclosure practices likely shaped participants’ narratives and may not have been fully captured in the original reports. Fourth, formal publication-bias testing is not applicable to qualitative meta-synthesis; nevertheless, selective publication and selective reporting remain possible, particularly because studies offering richer or more discussable accounts of sexuality may have been more likely to be published. Finally, many primary studies provided limited reflexive reporting on researchers’ positioning and influence, which reduced dependability in the ConQual assessment. Future qualitative work should therefore include broader language coverage, clearer subgroup reporting, and stronger reflexive and methodological transparency.

### 4.5 Clinical implications

This study has several important clinical implications. Sexual-health assessment should be systematically integrated into pre-treatment counselling, perioperative care, and long-term follow-up rather than being addressed only when patients report problems. Healthcare providers should adopt a couple-centred approach that actively involves partners in education, communication training, and dyadic coping support. A multidisciplinary sexual rehabilitation team comprising urologists, oncology and stoma-care nurses, psychologists, sex therapists, and pelvic-floor physiotherapists is needed to provide individualized care tailored to patients’ age, gender, treatment modality, relationship status, and cultural background. In addition, dedicated training programs should strengthen clinicians’ confidence, cultural sensitivity, and communication skills so that sexual concerns are recognized as a legitimate and routine component of bladder cancer care.

## 5 Conclusion

This systematic review and qualitative meta-synthesis integrated evidence from 10 studies and generated three synthesized findings that together provide a comprehensive picture of sexual distress and intimate relationship experiences among bladder cancer patients. The review shows that bladder cancer treatment can disrupt sexuality at physiological, psychological, relational, and existential levels, reshape couple relationships in different ways, and leave patients with substantial unmet sexual rehabilitation needs. These findings support the development of proactive, couple-centred, culturally sensitive, and multidisciplinary rehabilitation pathways that treat sexuality as a legitimate and essential component of bladder cancer survivorship care.
