# Supplementary material for: Sexual life and intimate relationship experience among bladder cancer patients: a systematic review and qualitative meta-synthesis
Source: Front Public Health. 2026 Aug 19;14:1887122. doi: 10.3389/fpubh.2026.1887122 (PMC13534141; doi:10.3389/fpubh.2026.1887122)
Supplement: Supplementary file 1 [file Table_1.DOCX]

**The supplementary material consists of one table:**

Table 1: The PubMed, Web of Science, Cochrane Library, Embase, CINAHL, CNKI, WanFang and Vip databases were systematically searched. The details retrieved for each database in the table.

Table1: The PubMed, Web of Science, Cochrane Library, Embase, CINAHL, CNKI, WanFang and Vip databases were systematically searched. The details retrieved for each database in the table.

| **PubMed** | | |
| --- | --- | --- |
| #1 | "urinary bladder neoplasms"[MeSH Terms] OR "bladder tumor*"[Title/Abstract] OR "bladder cancer*"[Title/Abstract]OR " malignant tumor of urinary bladder "[Title/Abstract] " urothelial  cancer "[Title/Abstract] OR " urothelial carcinoma "[Title/Abstract] | 88334 |
| #2 | " cystectomy "[MeSH Terms] OR " radical cystectomy "[MeSH Terms] OR " cystostomy "[MeSH Terms] OR " urinary diversion *"[Title/Abstract] OR " ileal conduit "[Title/Abstract] | 22059 |
| #3 | " sexual behavior "[MeSH Terms] OR " sexual attitude "[Title/Abstract] OR " sexual cognition "[Title/Abstract] OR " sexual life "[Title/Abstract] OR “sexual experience” [Title/Abstract] OR " sexual activity "[Title/Abstract] OR " sexual health"[Title/Abstract] OR " sexual function "[Title/Abstract] OR " sexual relationship "[Title/Abstract] OR " intimacy "[Title/Abstract] OR " intimate relationship "[Title/Abstract] OR " spouse* "[Title/Abstract] OR " sexual partner*"[Title/Abstract] | 182327 |
| #4 | "qualitative research"[MeSH Terms]OR "Qualitative"[Title/Abstract] OR qualitative study [Title/Abstract]"grounded theory"[Title/Abstract] OR "interviews as topic"[Title/Abstract] OR "focus group*"[Title/Abstract] OR OR"interview*"[Title/Abstract]OR "phenomenology*"[Title/Abstract] OR "ethnography*"[Title/Abstract] OR "case study"[Title/Abstract] OR "historic research"[Title/Abstract] OR "action research"[Title/Abstract] OR "experience*"[Title/Abstract] OR "view*"[Title/Abstract] OR "perspective*"[Title/Abstract] OR "perception*"[Title/Abstract] OR "attitude*"[Title/Abstract] | 2405484 |
| Qua | #1 AND #2 AND #3 AND #4 | 104 |
| **Web of science（Core set）** | | |
| #1 | TS=(urinary bladder neoplasms" OR bladder tumor* OR  bladder cancer* OR malignant tumor of urinary bladder OR urothelial cancer OR urothelial carcinoma ) | 182921 |
| #2 | TS=( cystectomy OR radical cystectomy OR cystostomy OR urinary diversion OR ileal conduit) | 47692 |
| #3 | TS=( sexual behavior OR sexual attitude OR sexual cognition OR sexual life OR sexual experience OR sexual activity OR sexual health OR sexual function OR sexual relationship OR intimacy OR intimate relationship OR spouse* OR " sexual partner*) | 1327007 |
| #4 | TS=(Qualitative OR grounded theory OR interviews as topic OR nursing methodology research OR focus group* OR interview* OR phenomenology* OR ethnography* OR case study OR historic research OR action research OR experience* OR view* OR perspective* OR perception* OR attitude*) | 14437960 |
| #5 | #1 AND #2 AND #3 AND #4 | 774 |
| **Cochrane** | | |
| #1 | MeSH descriptor: [bladder cancer] explode all trees | 2144 |
| #2 | (urinary bladder neoplasms" OR bladder tumor* OR  bladder cancer* OR malignant tumor of urinary bladder OR urothelial cancer OR urothelial carcinoma):ti,ab,kw | 7619 |
| #3 | #1 OR #2 | 7688 |
| #4 | MeSH descriptor: [cystectomy] explode all trees | 573 |
| #5 | (cystectomy OR radical cystectomy OR cystostomy OR urinary diversion OR ileal conduit):ti,ab,kw | 2705 |
| #6 | #4 OR #5 | 2742 |
| #7 | MeSH descriptor: [Sexuality] explode all trees | 1333 |
| #8 | (sexual behavior OR sexual attitude OR sexual cognition OR sexual life OR sexual experience OR sexual activity OR sexual health OR sexual function OR sexual relationship OR intimacy OR intimate relationship OR spouse* OR " sexual partner*OR):ti,ab,kw | 54655 |
| #9 | #7 OR #8 | 55055 |
| #10 | MeSH descriptor: [Qualitative Research] explode all trees | 1942 |
| #11 | MeSH descriptor: [Grounded Theory] explode all trees | 25 |
| #12 | MeSH descriptor: [Interviews as Topic] explode all trees | 2278 |
| #13 | MeSH descriptor: [Nursing Methodology Research] explode all trees | 302 |
| #14 | MeSH descriptor: [Focus Groups] explode all trees | 923 |
| #15 | (Qualitative OR grounded theory OR interviews as topic OR nursing methodology research OR focus groups OR focus group OR interviews OR interview OR phenomenolog* OR ethnograph* OR case study OR historic research OR action research OR experience* OR view* OR perspective* OR perception* OR attitude*):ti,ab,kw | 434944 |
| #16 | #10 OR #11 OR #12 OR #13 OR #14 OR #15 | 434944 |
| #27 | #3 AND #6 AND #9 AND #16 | 64 |
| **Embase** | | |
| #1 | 'bladder tumor'/exp | 124414 |
| #2 | 'bladder cancer'/exp | 98281 |
| #3 | 'bladder tumor':ti,ab,kw OR 'bladder cancer':ti,ab,kw OR 'urinary bladder neoplasm':ti,ab,kw OR ' malignant tumor of urinary bladder ':ti,ab,kw OR ' urothelial cancer *':ti,ab,kw OR ' urothelial carcinoma ':ti,ab,kw | 96922 |
| #4 | #1 OR #2 OR #3 | 146861 |
| #5 | 'radical cystectomy '/exp | 4354 |
| #6 | ' cystectomy '/exp | 41781 |
| #7 | ' radical cystectomy ':ti,ab,kw OR ' cystectomy ':ti,ab,kw OR ' cystostomy ':ti,ab,kw OR ' urinary diversion ':ti,ab,kw OR ' ileal conduit ':ti,ab,kw | 40912 |
| #8 | #5OR #6 OR #7 | 54775 |
| #9 | 'sexuality'/exp | 365222 |
| #10 | 'intimate relationship'/exp | 2984 |
| #11 | ' sexual behavior ':ti,ab,kw OR ' sexual attitude ':ti,ab,kw OR ' sexual cognition ':ti,ab,kw OR ' sexuality':ti,ab,kw OR ' sexual life ':ti,ab,kw OR ' sexual experience ':ti,ab,kw OR ' sexual activity ':ti,ab,kw OR ' sexual health ':ti,ab,kw OR ' sexual function ':ti,ab,kw OR ' sexual relationship ':ti,ab,kw OR ' intimacy ':ti,ab,kw OR ' intimate relationship':ti,ab,kw OR ' spouse ':ti,ab,kw OR ' sexual partner':ti,ab,kw | 145955 |
| #12 | #9 OR #10 OR #11 | 416737 |
| #13 | 'qualitative research'/exp | 163027 |
| #14 | 'grounded theory'/exp | 21498 |
| #15 | 'interviews '/exp | 537371 |
| #16 | 'nursing methodology research'/exp | 14845 |
| #17 | 'focus groups'/exp | 50514 |
| #18 | 'qualitative':ti,ab,kw OR 'grounded theory':ti,ab,kw OR 'interviews as topic':ti,ab,kw OR 'nursing methodology research':ti,ab,kw OR 'focus groups':ti,ab,kw OR 'focus group':ti,ab,kw OR 'interview':ti,ab,kwOR 'phenomenology':ti,ab,kw OR 'ethnography':ti,ab,kw OR 'case study':ti,ab,kw OR 'historic research':ti,ab,kw OR 'action research':ti,ab,kw OR 'experience':ti,ab,kw OR 'view':ti,ab,kw OR 'perspective':ti,ab,kw OR 'perception':ti,ab,kw OR 'attitude':ti,ab,kw | 3169436 |
| #19 | #20 OR #21 OR #22 OR #23 OR #24 OR #25 | 33444465 |
| #20 | #6 AND #11 AND #19 AND #26 | 169 |
| **CINAHL** | | |
| S1 | (MM"bladder cancer") | 11322 |
| S2 | (MM " urinary bladder neoplasms ") | 7568 |
| S3 | (MM "bladder tumor") | 6550 |
| S4 | SU(urinary bladder neoplasms" OR bladder tumor* OR  bladder cancer* OR malignant tumor of urinary bladder OR urothelial cancer OR urothelial carcinoma) | 22450 |
| S5 | S1 OR S2 OR S3 OR S4 | 12568 |
| S6 | (MM " cystectomy ") | 25927 |
| S7 | (MM " radical cystectomy ") | 6163 |
| S8 | (MM " cystostomy ") | 4613 |
| S9 | SU (cystectomy OR radical cystectomy OR cystostomy OR urinary diversion OR ileal conduit ) | 28872 |
| S10 | S6 OR S7 OR S8 OR S9 | 37481 |
| S11 | (MM "Sexuality") | 15183 |
| S12 | (MM " intimate relationship ") | 688 |
| S13 | (MM "Sexual function") | 1404 |
| S14 | (MM "Sexual relationship") | 2569 |
| S15 | (MM "sexual behavior OR sexual attitude OR sexual cognition OR sexual life OR sexual experience OR sexual activity OR sexual health OR sexual function OR sexual relationship OR intimacy OR intimate relationship OR spouse* OR " sexual partner*) | 20573 |
| S16 | S11 OR S12 OR S13 OR S14 OR S15 | 30380 |
| S17 | (MM "Qualitative Studies+") | 10882 |
| S18 | (MM "Grounded Theory") | 785 |
| S19 | (MM "Semi-Structured Interview") | 210 |
| S20 | (MM "Research Methodology+") | 138750 |
| S21 | (MM "Focus Groups") | 956 |
| S22 | SU ( Qualitative OR grounded theory OR interviews as topic OR nursing methodology research OR focus groups OR focus group OR interviews OR interview OR phenomenolog* OR ethnograph* OR case study OR historic research OR action research OR experience* OR view* OR perspective* OR perception* OR attitude* ) OR AB ( Qualitative OR grounded theory OR interviews as topic OR nursing methodology research OR focus groups OR focus group OR interviews OR interview OR phenomenolog* OR ethnograph* OR case study OR historic research OR action research OR experience* OR view* OR perspective* OR perception* OR attitude* ) | 1508863 |
| S23 | S17 OR S18 OR S19 OR S20 OR S21 OR S22 | 1799137 |
| S27 | S5 AND S10 AND S16 AND S23 | 158 |
| **CNKI** | | |
|  | SU=膀胱癌 +膀胱肿瘤 AND SU=膀胱切除术 +全膀胱切除 +膀胱癌根治术 +膀胱造口 +膀胱造瘘 +尿路造口 +泌尿造口 +尿流改道 AND SU=性关系 +性生活 +性功能 +性活动 +性态度 +性观念 +性体验 +性认知 +性困扰+性健康 +性伴侣 +亲密关系+亲密度AND SU=质性研究+扎根理论+现象学+描述性+行动研究+历史研究+民族志+人种学+个案研究+访谈+混合研究 | 35 |
| **WanFang** | | |
|  | 主题:(“膀胱癌 +膀胱肿瘤”) AND 主题:( “膀胱切除术 +全膀胱切除 +膀胱癌根治术 +膀胱造口 +膀胱造瘘 +尿路造口 +泌尿造口 +尿流改道”) AND 主题:(“性关系 +性生活 +性功能 +性活动 +性态度 +性观念 +性体验 +性认知 +性困扰+性健康 +性伴侣 +亲密关系+亲密度) AND 主题:(“质性研究”+“扎根理论”+“现象学”+“描述性”+“行动研究”+“历史研究”+“民族志”+“人种学”+“个案研究”+“访谈”+混合研究) | 20 |
| **Vip** | | |
|  | (题目或关键词=(膀胱癌 +膀胱肿瘤 ) AND 题目或关键词=（膀胱切除术 +全膀胱切除 +膀胱癌根治术 +膀胱造口 +膀胱造瘘 +尿路造口 +泌尿造口 +尿流改道）AND题目或关键词=（性关系 +性生活 +性功能 +性活动 +性态度 +性观念 +性体验 +性认知 +性困扰+性健康 +性伴侣 +亲密关系+亲密度）AND AND (题目或关键词=(质性研究+扎根理论+现象学+描述性+行动研究+历史研究+民族志+人种学+个案研究+访谈+混合研究) | 13 |
